# From Design to Deployment: Decentralized Coordination of Heterogeneous Robotic Teams

**DOI:** 10.3389/frobt.2020.00051

**Published:** 2020-05-07

**Authors:** David St-Onge, Vivek Shankar Varadharajan, Ivan Švogor, Giovanni Beltrame

**Affiliations:** ^1^INIT Robots Laboratory, Department of Mechanical Engineering, École de technologie supérieure, Montreal, QC, Canada; ^2^MIST Laboratory, Department of Computer Engineering and Software Engineering, Polytechnique Montreal, Montreal, QC, Canada

**Keywords:** decentralized behaviors, swarm intelligence, heterogeneous robotic teams, over-the-air update, swarm systems, control framework, swarm programming

## Abstract

Many applications benefit from the use of multiple robots, but their scalability and applicability are fundamentally limited when relying on a central control station. Getting beyond the centralized approach can increase the complexity of the embedded software, the sensitivity to the network topology, and render the deployment on physical devices tedious and error-prone. This work introduces a software-based solution to cope with these challenges on commercial hardware. We bring together our previous work on Buzz, the swarm-oriented programming language, and the many contributions of the Robotic Operating System (ROS) community into a reliable workflow, from rapid prototyping of decentralized behaviors up to robust field deployment. The Buzz programming language is a hardware independent, domain-specific (swarm-oriented), and composable language. From simulation to the field, a Buzz script can stay unmodified and almost seamlessly applicable to all units of a heterogeneous robotic team. We present the software structure of our solution, and the swarm-oriented paradigms it encompasses. While the design of a new behavior can be achieved on a lightweight simulator, we show how our security mechanisms enhance field deployment robustness. In addition, developers can update their scripts in the field using a safe software release mechanism. Integrating Buzz in ROS, adding safety mechanisms and granting field updates are core contributions essential to swarm robotics deployment: from simulation to the field. We show the applicability of our work with the implementation of two practical decentralized scenarios: a robust generic task allocation strategy and an optimized area coverage algorithm. Both behaviors are explained and tested with simulations, then experimented with heterogeneous ground-and-air robotic teams.

## 1. Introduction

The range of applications for multi-robot systems is constantly and rapidly expanding. Small groups of heterogeneous robots collaborating to extend their individual potential were repeatedly proven to be successful (Rekleitis et al., 2001; Kruijff et al., 2012; Lliffe, 2016). Unfortunately, each unit of these scenarios is necessary, to the point that a single failure will most likely cause the mission to fail. By leveraging a greater number of similar agents, individual failures can be compensated, while the imprecision of sensors can be mitigated by fusion of multiple sources. Swarm robotics has been known for decades to be a possible solution to many problems in dynamic, hostile, and unknown environments (Brambilla et al., 2013). A Swarm Robotics System (SRS) must be flexible, scalable, and robust (Şahin, 2004). Unfortunately, swarm robotics requires development tools specific to decentralized systems that are still hardly available.

Researchers are very active in developing behaviors for robotic swarms (Bamberger et al., 2006; Brunet et al., 2008; Hauert et al., 2011; Bayindir, 2016; Davis et al., 2016), with support from a handful of companies and some open source initiatives (Goc et al., 2016; Pickem et al., 2016). These affordable platforms grant access to physical implementation with a significant number of robots, but lack a set of software tools for the implementation of their collective behavior. Furthermore, swarms share common behavioral paradigms: no predefined roles, and control based on local interactions. For a swarm system, and in particular one with heterogeneous members, communication, neighbor management, and data sharing need to be re–implemented for each platform and experiment. For instance the work presented in Hauert et al. (2011), similar to many of the previously mentioned ones, is hardware specific and cannot be ported to other robotic systems easily.

The development of an optimized and specialized software infrastructure, one that is sufficiently flexible to make robotics researchers feel unconstrained, while simultaneously increasing their development efficiency is a tedious, and often unsuccessful, task. ROS has established itself as a standard for robot development, but the community is still exploring the challenges of swarm engineering (Davis et al., 2016). This issue became more apparent with the introduction of programming languages that are specific for swarm development (Bachrach et al., 2010; Pinciroli et al., 2015).

Among those, Buzz is a domain-specific programming language for robot swarms (Pinciroli and Beltrame, 2016). Its purpose is to help researchers and practitioners by providing a set of primitives which accelerates the implementation of swarm-specific behaviors. Buzz comes with an optimized virtual machine that runs on all swarm members, and each robot executes a common program or script. The main peculiarity of Buzz is that it merges bottom–up behavior development with a top–down strategy definition for the whole swarm (Crespi et al., 2008). Buzz and its virtual machine allow a script to be deployed on any autonomous robot: from small desk robots, to Unmanned Air Vehicles (UAVs) and Unmanned Ground Vehicles (UGVs) of any size, and even satellites. While Buzz was natively deployed on embedded systems (Kilobots, Zooids, and Kheperas robots)^1^, larger robots require integration within a software ecosystem that allows roboticists to interface with different sensors, actuators, and complex algorithms easily.

To address this issue we introduce ROSBuzz, the ROS implementation of the Buzz Virtual Machine (BVM). Much more than an adapter or a facade, it enables (a) fast script–based programming of complex behaviors, (b) seamless script porting on different hardware, (c) safe field deployment, (d) over-the-air updates on the field, and most importantly (e) it allows a coherent design flow from simulation to field deployment. While (a) and (b) arise from the integration of Buzz in the ROS ecosystem, (e) is possible only through ROSBuzz and its other core contributions (c) and (d). To present ROSBuzz we first recall the key primitives of Buzz from (Pinciroli and Beltrame, 2016) (section 2.1), then explain the details of this ROS node architecture (section 2.2), its specific simulation-to-field workflow (section 2.3), and the integrated mechanisms to minimize risk at deployment (section 2.4). To test the ROSBuzz performance we introduce two decentralized behaviors: a task allocation strategy (section 3.1), and an area coverage algorithm (section 3.2). Both algorithms are assessed in term of robustness to packet loss and scalability in simulation and with real world experiments.

## 2. Methods

In academia and industry, ROS has become a *de facto* standard for any serious mobile robotics application. The sheer number of community-developed tools (e.g., Rviz^2^, Rqt Graph^3^, PlotJuggler^4^, etc.) makes it almost indispensable for developing, testing and integrating all the software layers of the autonomy stack that a single robot requires. Though ROS can be used to simultaneously control multiple robots, it was never really designed for decentralized coordination of robotic teams.

The long-awaited ROS 2.0 is welcome by the multi-robot research community: it brings solutions for some known issues in controlling multi-robot systems. Mostly, those are related to networking, real-time processing, and defining relationships between robots, but the challenges of decentralized multi-robot systems still remain: the main purpose of ROS 2.0 is to provide transparency of the network layer, while control resides in the implementation of the swarm behavior.

The main differences and challenges of controlling a swarm, as opposed to a single robot, are that by definition swarms must be (a) decentralized, and (b) programmed with the same behavior.

(a) means that there cannot be a central point or a single robot in charge of defining the behavior of the swarm. However, this does not mean that any member of the swarm cannot take specific roles, which brings us to (b). The robots cannot be programmed as individuals, or in technical terms, the code deployed to each member of the swarm must be identical. Therefore, in the true spirit of the swarm behavior, the implementation must be such that all the members of the swarm contain the same code, but the behavior of the individual is defined and directed by the entire group and the environmental context.

### 2.1. Buzz, Swarm Language, and Virtual Machine

To better understand our implementation choices, please consider the following factors which generally define a swarm: (a) decentralized decision making, (b) behavior defined on local interactions and environmental context, and (c) information propagation latency. Buzz grants the developer with premises and constructs that ease the deployment of top–down swarm strategies: a set of rules regulating the swarm members actions following their interaction and the mission's goals. This aspect is core to most swarm intelligence algorithms developed in the past decades. However, real robotic systems benefit in many contexts from the heterogeneity of their abilities (for instance different sensors and locomotion modes). Buzz allows to program for sub-swarms, i.e., subset of the swarm with specific attributes to complete specific type of tasks (Pinciroli and Beltrame, 2016).

#### 2.1.1. The Buzz Toolbox

Buzz provides literals and data structures to address three key concepts in defining a swarm behavior^5^:

- *Virtual stigmergy (VS)*: a bio–inspired shared tuple space. The original concept of stigmergy is an environment–mediated communication modality used by social insects to coordinate activity (Camazine et al., 2003). VS is implemented as a shared memory table containing 〈key,value〉 pairs. The shared memory table stored in a local copy on each robot, which is synchronized via communication only when needed. Each 〈key,value〉 tuple is associated with a timestamp [a Lamport clock Lamport, Lamport] and the ID of the last robot that modified the data. Tuples and metadata are shared between swarm members via a gossip algorithm (Pinciroli et al., 2016). Each robot locally decides when to re-broadcast information based on the timestamp and conflict detection and resolution mechanisms. Overall, robots always converge to a common set of tuples. The details of the inner workings of the Virtual Stigmergy can be found in a previous publication (Pinciroli et al., 2016).
- *Swarm Aggregation*: is a literal which allows for grouping of robots into sub–swarms, through the principle of dynamic labeling (Pinciroli and Beltrame, 2016). The swarm construct is used to create a group of robots that can be attributed with a specific behavior, which differs from the other robots, based either on the task or robot abilities.
- *Neighbors Operations*: in Buzz refer to a rich set of functions (reduce, map, size, foreach, broadcast, listen, etc.) which can be performed with or on neighboring robots through situated communication (Støy, 2001). Neighbors are defined from a network perspective as robots which have a direct communication link with each other. With situated communication, whenever a robot receives a message, the origin position of the message is also known to the receiver.

These primitives constitute essential functionality that comes with Buzz and enables robotics software engineers to accelerate their way to developing swarm behaviors. To demonstrate this consider the following code:

var accum = neighbors.map(lj_vector).reduce(lj_sum, math.vec2.new(0.0, 0.0)).

With this line, every robot in the swarm uses a neighbors structure to map a certain function to all the elements of the list (i.e., neighboring robots) and uses a rolling computation to reduce it to a single value used by a robot^6^. By rolling computation, we refer to the fact that reduce applies the function lj_sum to each neighboring robot's relative position to obtain a single value (accum) as per the logic defined in the lj_sum function.

Buzz is an extension language: if a user needs a specific primitive not provided by its current syntax, it can be easily added using C code. In fact, Buzz provides an intuitive way to expose any function written in C to the Buzz script, with access to the current execution context, i.e., C functions can access the literals and data sets used in the script.

A Buzz script is compiled into a memory-efficient and platform-agnostic bytecode to be executed on the Buzz Virtual Machine (BVM). To interface the BVM with the robots' actuators and sensors, we use ROS. The following section describes how ROS and Buzz are integrated to allow seamless and platform-agnostic execution and extension of Buzz scripts that define swarm behavior.

### 2.2. ROSBuzz

ROS is a widely used tool, accepted by both researchers and professionals as it improves the productivity and compatibility of robotics development, while Buzz provides essentials for designing and developing swarm behaviors.

A ROS node is generally an executable that uses ROS^7^ to communicate with other nodes. ROSBuzz puts Buzz and ROS together, providing a ROS node which encapsulates the Buzz Virtual Machine. Furthermore, we implement communication between swarm members with the Micro-Air Vehicle Link (MAVLink) protocol, which is widely available through the MAVROS implementation. Buzz messages are serialized and packed into the MAVLink standard payload messages, while the ROSBuzz node provides the BVM with access to these messages. As such, ROSBuzz allows any MAVLink-capable mobile robot to join the swarm, using the common behavior defined by the *buzz script* provided by the *ROS launch file*.

**Figure 1:**
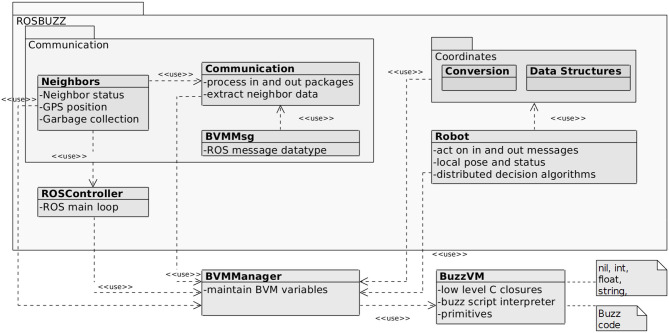
Simplified class diagram of the ROSBuzz software architecture: step-based BVM (lower right) integrated to the event-based ROS ecosystem.

The software architecture of the ROSBuzz node (shown in Figure 1) is organized in three layers which reconcile the step–based (sense, plan, act) execution nature of Buzz and the event–based nature of ROS.

The namespace ROSBuzz represents the entire ROS package. Upon launching, ROSBuzz initializes the main ROS loop and the necessary configuration parameters. Those consist of ROS callback functions for subscribers, publishers, and services which hold references to MAVROS specific messages and expose them to the BVM. These messages can then be used for sensing, planning and acting.

On top of Figure 1, the main namespace ROSBuzz contains two additional namespace: Communication and Coordinates. The latter is used to implement various data structures that represent positions in coordinate systems with different bases and the transformations between those. The Communication namespace is used to process the MAVROS payload and extract the information about the robots neighbors. Namely, BVMMsgs defines the ROS messages for the MAVROS communication, the Communication class processes the incoming and outgoing messages, while Neighbors is a class used to store neighbor information.

There are two additional classes within the ROSBuzz namespace: ROSController, which defines the ROS node containing the main loop and implements the callback references; and the Robot class, which implements some logic and stores local information about the robot.

To use this software stack, a user needs to install a ROSBuzz package, write a Buzz script, and point the ROS launch file to it. The BuzzVM interprets and executes the script, and executes the following loop: (a) process incoming messages, (b) update sensors information, (c) perform a control step, (d) process outgoing messages; and finally (e) update the actuation commands. The BVMManager class is used to mediate the step-based nature of the Buzz and event-based nature of ROS. As messages come in the main ROS loop (in the ROSController class), the BVMManager makes the latest information available to the BuzzVM interpreter.

#### 2.2.1. Under the Hood

**Figure 2:**
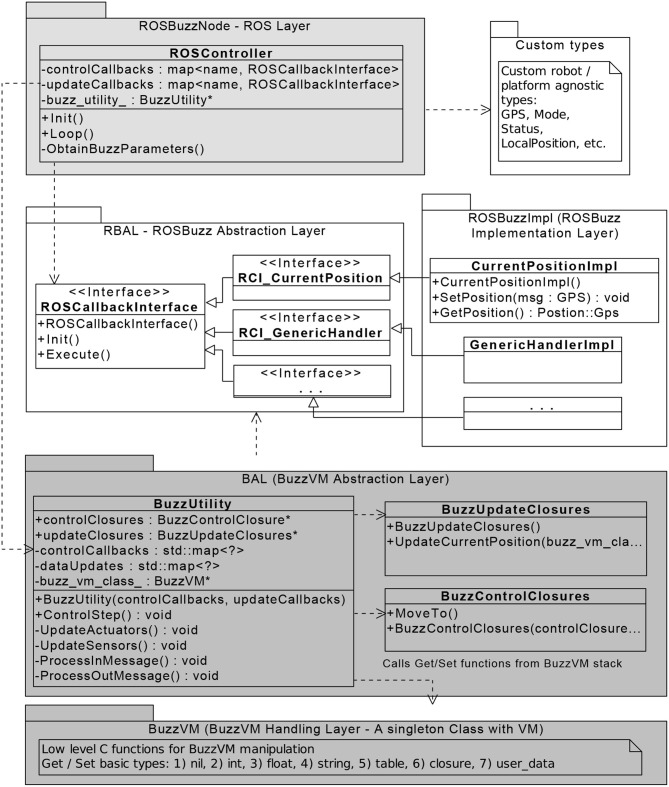
Relationship between classes of the internal ROSBuzz software architecture.

To extend Buzz, a designer needs only to use C to expose additional functionality to the Buzz script. In other words, it is possible to use existing libraries or algorithms from any other language outside of the BVM and still grant access to these functionalities inside a script. A C function can be exposed as a Buzz closure, which is bound and registered to the BuzzVM, making the closure available for use in the Buzz script. To provide more details in how Buzz and ROS actually work together, let us consider Figure 2 and the following scenario: the Buzz script needs to access the latest positional information of the neighbors to avoid a collision. For this scenario, the control flow is as follows.

Upon starting the ROSBuzz node, the update and control callbacks are initialized and the main ROS loop starts. In each step, the main loop calls a ControlStep function from a BuzzUtility instance. After processing all the *in messages* from the neighboring swarm members, the UpdateSensors function delivers the information about the current position to BuzzVM using the UpdateCurrentPosition function (which uses the updateClosures collection, which in turn provides access to the Execute function of the CurrentPositionImpl). With this setup, the Buzz script can access the position information during the execution of its ControlStep. However, to send actuation information back to ROS, after the ControlStep method, the main ROS loop calls the UpdateActuators which in the similar way uses the BuzzControlClosures to perform actuation via ROS callback functions.

From a software engineering standpoint, ROSBuzz provides certain level of abstractions to make it maintainable, upgradeable, and extendable. Figure 2 shows the abstraction layers within ROSBuzz, with which an user can independently adapt the implementation layer to fit the current needs without changing any other layers of the software. ROSBuzz provides robotics researchers and practitioners a turnkey system that can transform a heterogeneous group of robots into a swarm.

Furthermore, ROSBuzz provides the developer with a consistent simulation-to-deployment infrastructure, and mechanism to enhance the robustness of the deployment itself: over-the-air behavior updates and barrier consensus. The next sections detail these features.

### 2.3. Simulation to Deployment

Designing applications for delicate and expensive hardware puts a lot of pressure on the developers. When tens of robots are deployed to achieve a coordinated task, the risk of failure and hardware damage increase rapidly. To cope with this issue, a common approach is to carefully simulate the control before taking it out to the field. ROSBuzz also provides a step-by-step workflow to minimize the risks of deploying decentralized behaviors: a low-resource simulator to iterate quickly on the design and test thousands of units, followed by a more realistic full-stack software-in-the-loop environment, extended whenever available to hardware-in-the-loop validation and finally to the deployment of the behavior in the field.

**Figure 3:**
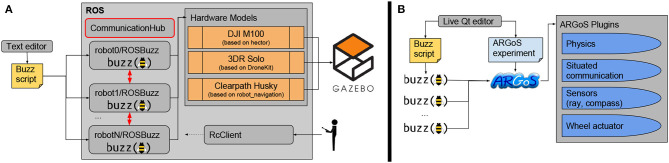
Simulation environments: **(A)** with Gazebo and ROS nodes, allowing for accurate dynamics and operator inputs, and **(B)** with ARGoS to simulate large swarm size in a lightweight environment.

Figure 3 shows the modules and ecosystem of both simulation setups. Since the early development phases of Buzz, the BVM was integrated in the ARGoS simulator (Pinciroli et al., 2012), which features a Buzz editor, allowing for quick development and integration of behavioral scripts (Figure 3B). While ARGoS can support thousands of units, it does not accurately represent the dynamics of the robots, it is not compliant with the ROS architecture, and does not allow external control during mission operations. Therefore, we added a second simulation stage based on ROS and Gazebo (Figure 3A), leveraging community packages available for ROS. Three hardware adapters are currently provided for Gazebo using the hector package (Meyer et al., 2012) for the Matrice 100, the DroneKit-SITL for the 3DR Solo, and the nodes provided by Clearpath for the Husky rover. Multiple instances of ROSBuzz are launched in a separate group namespace, alongside their hardware emulators. On a Core i7 laptop equipped with a NVIDIA graphics card, we are able to smoothly simulate 50 DJI Matrice 100 in Gazebo. The inter-robot communication is managed by a relay node that acts as a communication hub between ROSBuzz instances. The relay provides control over the communication simulation parameters with user-defined packet drop rates, latency, bandwidth, and communication range. In section 3, we use this simulation ecosystem to show the scalability of our system and its robustness to packet loss.

### 2.4. Robustness-Enhancing Mechanisms

The tools introduced in the above sections grant the developers with a software ecosystem easing the implementation, simulation and deployment of decentralized behaviors. This is at the core of the apparent needs in multi-robots team technology, but entirely rely on the developer to ensure minimal risk at deployment. To help the user enhance the robustness of the behavior in the field, we integrate common safety mechanisms (described in section 3.2.3) and we provide two essential contributions with ROSBuzz: a consensus strategy referred to as “barrier” and a safe Over-The-Air (OTA) script update mechanism.

#### 2.4.1. Barrier

When dealing with the coordination of multiple robots, a group of robots that comes to an agreement on the value of some variable, is said to have reached *consensus*. One of the key elements for designing complex swarm behaviors, is the ability to create swarm-level state machines, where all robots agree on the current state of the swarm (or sub-swarm). For this purpose, we designed a *barrier* mechanism, which allows the synchronous transition of all swarm members from one state to another. The swarm construct of Buzz also broadens the use of the barrier on specialized subswarms; a handy feature to split the group over parallel missions. A safe waiting state (idle, hover) is used to wait for all robots to agree on the following state. The barrier uses a VS table (section 2.1): each robot updates a state value associated to its own unique id when it is ready to change state (i.e., behavior). The robot IDs are attributed following the network interface address (for instance Xbee serial number or WiFi IP address). Consensus is reached when the table size equals the swarm size and all values correspond to the same outgoing state: then all robots can transition to the next behavior.

The Buzz functions implementing the barrier are detailed in Listing 1. barrier_create is called once and barrier_wait at each step, until trans_st or resume_st are called, stopping the barrier loop.

**Listing 1:**
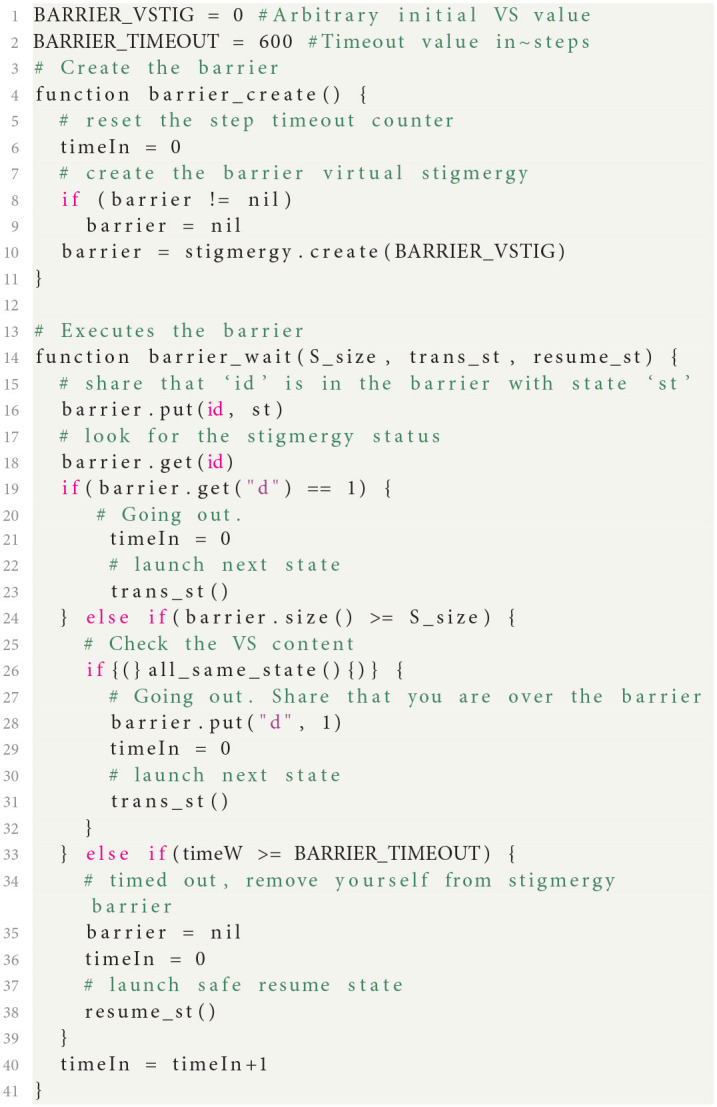
Barrier implementation of consensus in Buzz language.

We first create the barrier data structure: we initialize a VS table with a unique key (BARRIER_VSTIG). Then at each step of the BVM, the function barrier_wait is called with a transition state and a resume state. At each step, the robot puts its state in the VS table with its ID as a key. The robot then checks the table size: if it reaches the swarm size, the robot checks all values to ensure every unit is ready to go to the next state. If they are, the robot transitions its state and pushes a new value, d, in the table so the others know the barrier is done without checking all states. Otherwise, after a timeout (timeIn equals BARRIER_TIMEOUT), the robot resumes its previous behavior. We acknowledge that such a barrier mechanism can impact the scalability of any swarm algorithm deployed with our infrastructure. For this reason, the first experiment presented below (section 3.1) assesses the performance and usage of the barrier.

#### 2.4.2. Update Mechanism

**Figure 4:**
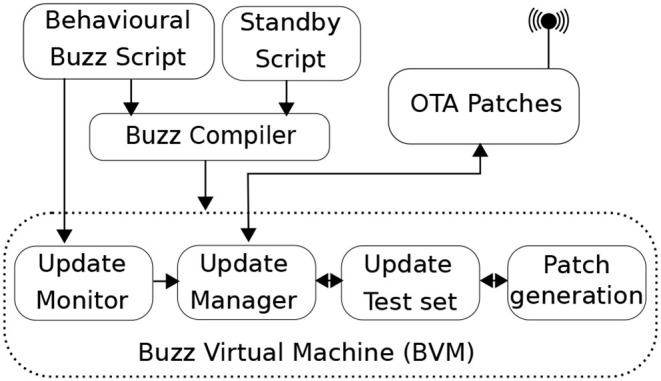
Update monitor within the Buzz Virtual Machine.

ROSBuzz provides a mechanism to hot swap the behavior script of the robots safely, with rollback strategies in case of update failures. The need for a reliable in-mission script update arises quickly when developing and experimenting with new behaviors. Since all robots in the swarm run the exact same script, they need to update simultaneously and ensure they stay coordinated. Our Over-The-Air (OTA) mechanism gets triggered either by modifying the script file or by a neighbor propagating a new behavior patch. The components of the update mechanism are summarized in Figure 4: the update monitor watches for changes in the script file and notifies the update manager of changes; then the new changes are compiled, tested, and an encrypted binary patch is generated, to be sent to all the other robots. The newly generated code version (id) is propagated through gossip based broadcasts, and when a version mismatch is identified by a robot, this latter requests the patch to its neighbors. To ensure safe transitions across versions, the robots switched to a standby behavior (a platform-dependent safe state) and a barrier is initialized to reach consensus on the code version to use on the swarm. For more information on this update mechanism, we refer the reader to the exhaustive presentation in (Varadharajan et al., 2018). The mechanism performance is closely dependent of the network quality. We tested our update system extensively with WiFi and Xbee mesh networks, and proved its convergence even in extremely poor network conditions in (Varadharajan et al., 2018).

## 3. Experiments

As previously stated, the software ecosystem is platform-agnostic. Any robot can have a MAVROS-compatible driver node and a peer-to-peer communication network (WiFi-based, Xbee/Zigbee, etc.). The rest of the paper presents the deployment of two decentralized behaviors, from design to field deployment, passing through simulation.

ROSBuzz, alongside our custom Xbee manager node (XbeeMav), was deployed on NVIDIA boards (TK1, TX1, and TX2), all running Ubuntu (16.04 or 18.04) for onboard control of a fleet of DJI Matrice 100 quadrotors (M100). DJI provides an onboard SDK that can be interfaced in ROS to be compliant with MAVROS. Using the same NVIDIA boards, we deployed ROSBuzz on a fleet of Pleaides Robotics Spiri quadcopters.

We also integrated a smaller and more resource-limited platform: the 3DR Solos, running ROSBuzz on Raspberry Pis 3 with Raspbian. As long-demonstrated by the ArduPilot community, the MAVlink protocol is also perfectly fitted to command and monitor rovers^8^. ROSBuzz was thus ported to a Clearpath Husky to control its navigation within an heterogeneous ground to air swarm. All robots use a Xbee 900 Pro module for inter-robot communication. The serialized and optimized Buzz messages payloads are transferred through a MAVlink standard payload message (64 b array).

We conducted two outdoor experiments with backup pilots for each robot: an autonomous task allocation demonstration with Solos, Husky and M100 (section 3.1), and a user driven area coverage demonstration with Spiris and M100 (section 3.2).

### 3.1. Task Allocation

A common scenario for a robot group is to execute a given queue of tasks evolving throughout the mission. Before optimizing the allocation of the tasks, the swarm must have a mechanism to ensure it will reach consensus on a given set of allocations. To ease the behavior visualization, let us represent the tasks with target positions in the following description. This demonstration is adapted from the work of (Li et al., 2019), where the authors progressively place robots in a formation starting from a root robot selecting neighbors to be placed in the formation and proceeding recursively, with the newly placed robots selecting more followers and so on until the formation is complete. We integrated our barrier mechanism in (Li et al., 2019)'s state machine, deployed the algorithm in ROSBuzz over a real decentralized network [Xbee, as opposed to an emulation using a communication hub and WiFi in Li et al., 2019] on UAVs (instead of wheeled indoor platforms), and we used the algorithm for task allocation instead of graph formation.

#### 3.1.1. Algorithm

We assume that all robots involved in the mission are aware of the list of tasks and their location. This hypothesis is not limiting since the structure table can be shared before the robots' deployment or through run-time broadcast using VS (refer to section 2.1.1 for a definition).

This table contains spatial coordinates of each the tasks. However, robots are not pre-assigned to a specified task in the mission. The behavior law allows them to find proper tasks through simple local interactions with other robots leveraging the Buzz neighbor structure (see section 2.1.1), including robots that are already part of the mission and robots that are not yet assigned a task. This process can drive free robots to participate in the mission gradually or, from the perspective of the mission, it will attract free robots to join, allowing it to grow dynamically.

**Figure 5:**
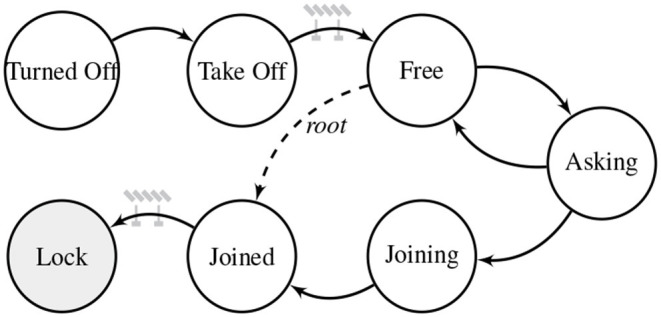
The behavior law of the progressive task allocation algorithm represented as a finite state machine. Every robot joining the mission will experience states *TurnedOff*, *TakeOff*, *Free, Asking, Joining* and *Joined*. Before switching to state *Free*, and *Lock* the robots wait for consensus in a transition barrier state.

The mission process starts when a robot gets the task 0 of the list. The progressive attribution of tasks will start from this robot, called the root, it is thus considered joined in the mission as soon as it goes out of the barrier following *TakeOff*, as shown with the dashed line in Figure 5. This unique robot has to be elected through interaction between the robots (i.e., a consensus) or a special robot can be attributed this role. The behavior law is represented as a finite state machine, shown in Figure 5. It consists of seven states: *Turned Off*, *Take Off*, *Free, Asking, Joining, Joined* and *Lock*. After a user asked to start the mission, the stakeholder, i.e., the drone the user is connected to, share the information for take off. The assignment of tasks will start only after the first barrier, waiting for all members to be at a safe height, hovering. In state *Free*, the robot will circle around the edge of the mission zone, namely the structure composed of *Joining* and *Joined* robots, and search for a proper task in the list, using only neighbor interaction (see section 2.1.1). When such a task is found, and both predecessors are within sight (from a network topology point-of-view), the *Free* robot will transit to state *Asking*, sending a message to request for the task. Once the request is approved by the *Joining* and *Joined* robots, the robot transits to state *Joining*. From that point on, it is part of the formation and is attributed a task (position) of the mission. With the knowledge of its *Joined* parent and of its own task position, the robot will compute its target GPS coordinates and navigate to it. Furthermore, since each robot needs only one predecessor (a robot already joined in the tree), it is not necessary to keep the entire structure of the mission, but rather only a predecessor tree.

This algorithm is a perfect fit for dynamic missions, i.e., changing number of robots and/or mission tasks. Its Buzz script is available online^9^ and was extensively tested in simulation (section 3.1.2) and in the field (section 3.1.3).

#### 3.1.2. Simulations

**Figure 6:**
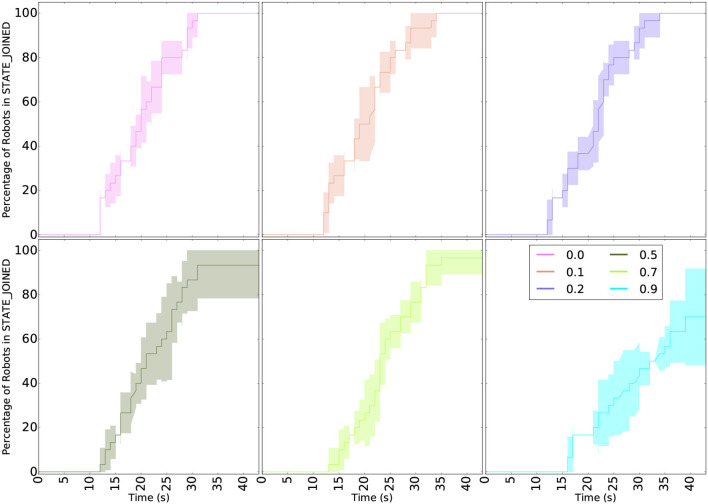
Robustness to packet drop of the task allocation algorithm presented in section 3.1.1. The curves show the percentage of robots in the last state of the algorithm: JOINED. Each scenario (packet drop rate) was ran 5 times and the variability of the results are shown in the area around the curves. All simulations were ran with 6 robots. The algorithm converges in most runs up to 70% packet loss.

Before releasing the behavior on field robots, we must assess its robustness to packet drop in the communication network. The task allocation algorithm is highly dependent on communication performance between peers in the swarm to reach consensus. The first set of simulations presented in Figure 6 test scenarios with up to 90% packet drop. It demonstrates the converge of the barrier mechanism used in the algorithm and its robustness to imperfect communication. The results of Figure 6 illustrate the time required by each robot to join a formation (i.e., reaching a JOINED state, as described in section 3.1.1). The conclusion is straightforward and expected: a higher drop rate requires more time to complete the mission. Without packet lost the simulated fleet converge in <30 s while with a packet drop rate of 90%, the robots take almost 45 s. For 20% packet loss or less, all robots converged in all runs. For 70 and 90%, on average more than 90% of the robots converged to their final state, with a minimum of 80%. As for 90% packet loss, a harsh condition that we never experienced in the field, only 70% of the robots converged on average, with a minimum as low as 50%, but still several runs showed 90% of the robots converging. Furthermore, if 90% packet loss can be observed in some rare cases, it is likely to not be continuous (since all robots are expected to be moving), as opposed to the constant drop rate used in these runs. These results show the high robustness of our algorithm and communication layers to communication issues. A simulation run with 30 robots is shown in the Supplementary Video.

The most significant output of this set of simulations is that consensus is always reached, even with large packet drop probability. The time required to reach JOINED state is not representative of the time required by the consensus mechanism alone since all robots have to move to their task to reach this state. Indeed, as explained in section 3.1.1, each task is associated with a target location and the robot will be considered to have joined the mission only when they reached it.

**Figure 7:**
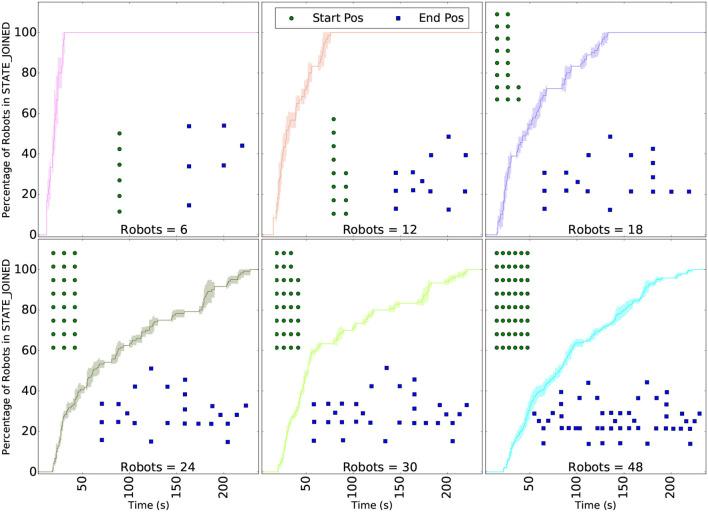
Scalability of the task allocation algorithm presented in section 3.1.1. The curves show the percentage of robots in the last state of the algorithm: JOINED. Each scenario (packet drop rate) was ran 5 times and the variability of the results are shown in the area around the curves. The green dots illustrate the launch position of the robots and the blue dots, their final position. All simulations were ran without packet lost. The algorithm converges even with 48 simulated robots.

As mentioned in the introduction, a swarm behavior is expected to be scalable, i.e., to behave in a similar way with both small and large robot teams without changes in the script (Pinciroli et al., 2016). Figure 7 shows simulations from 6 to 48 robots, and the time required for the robots to reach the JOINED state. For each scenario the task allocation, represented by a final formation, is different: 6 go to “P,” 12 to “PO,” 18 to “POL,” 24 to “POLY,” 30 to “PPOLY,” and 48 to “YLOPPOLY.” While 6 robots converge in <30 s, 48 robots take more than 200 s to reach their final formation. We must recall again that more distance is traveled by some robots, slowing down the convergence together with the increase of time for consensus between more robots.

#### 3.1.3. Field Deployment

We validated the task allocation algorithm in the field with small heterogeneous swarms: three M100, one or two Solos, and a Husky.

We conducted four outdoor experiments to test different topologies and formation geometries. Each mission had a different set of robots and different localized tasks, represented with four different graphs:

1. 2 branches (“L” shape) with 4 quadrotors and a rover,
2. 3 branches (“Y” shape) with 5 quadrotors and a rover,
3. 3 branches (“Y” shape) with 4 quadrotors and a rover,
4. 3 branches (“Y” shape) with 5 quadrotors only.

**Figure 8:**
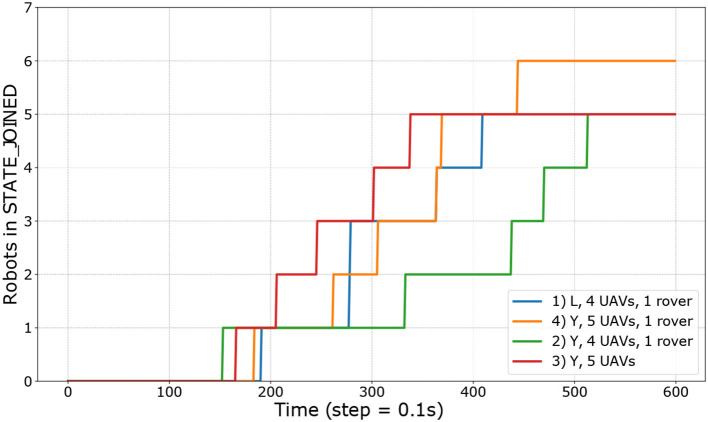
Time required for all the robots to join on each of the four experiments conducted. Four experiments had five robot while the orange one had six. To reach the STATE_JOINED, each robot has to reach the physical location of its task.

The time required for each unit to join the mission, i.e., to get its assigned task and move to its target position, is illustrated in Figure 8. The first robot to join takes more than 150 s to wait for the rest of the fleet to takeoff and get over the first barrier. As seen in the first experiment, some robots are parents (predecessor in the graph) to more than one subsequent task and make it possible to have two robots simultaneously joining the mission. In the last experiment, the first three robots joined in <250 s most likely because the ground-to-air communication in the other scenarios is slowing down the attribution of the tasks. The last experiment is shown in the Supplementary Video. Except for the third experiment, the average time to get a new robot to join is less than half a minute.

The time required to join is influenced by the network performance since each robot need to be assigned a label from its parent before moving. With Xbee 900 MHz, the range is large, but the low bandwidth and dropped packets can affect the performance.

**Figure 9:**
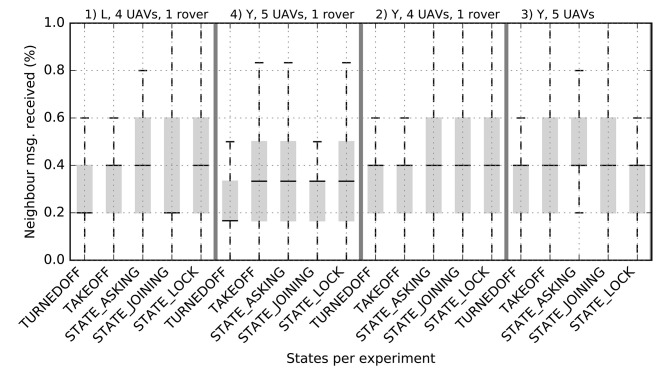
Average ratio of neighbors messages received over the total swarm member for different states.

Figure 9 shows the ratio of neighbor messages received over the swarm size. Indeed, in a Buzz step, each robot sends a message to all its neighbors sharing its position together with a payload relevant to the current step operations. We can observe that in average the *Turned Off* and *Take Off* states catches fewer messages than the other states. This can be explained with the radio wave deflection created by the irregularities of the ground.

**Figure 10:**
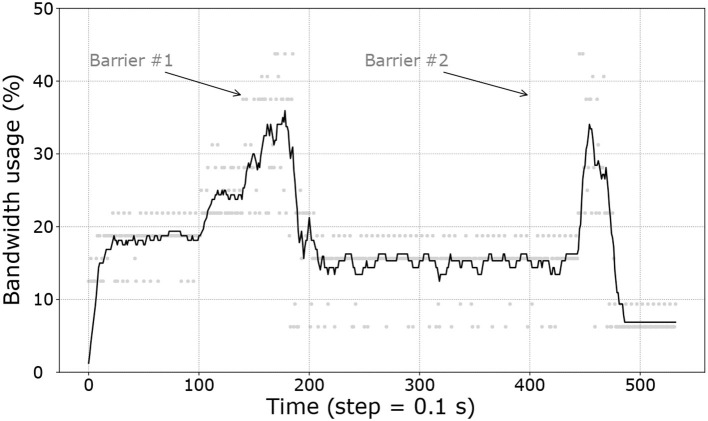
Moving average of the bandwidth usage based on a window of 30 samples (which are represented by gray dots).

Finally, Figure 10 shows the worst example of bandwidth usage for all robots on all experiments. It is clear that the maximum available payload per step, i.e., the Xbee frame size (250B, illustrated as a ratio), is never exceeded.

### 3.2. Semi-autonomous Exploration

In several application scenarios envisioned for robotic teams, they should not be fully autonomous: the operator expertise is mandatory to the success of the exploration mission. In post-disaster emergency response, for instance, successful missions highlighted the importance of collaborative and complementary work between human and robots, also known as a *coactive* approach (Szafir et al., 2017). A similar reasoning applies to many exploration missions in unknown and complex environments: in order to optimize the mission strategies under multi-objective pressure (geological analysis, mapping, specific ground feature search, etc.) a human expert is still required. The following demonstration is designed to let the user continuously monitor and control the swarm exploration mission.

#### 3.2.1. Algorithm

The Voronoi tessellation (Alexandrov et al., 2018) is an algorithm that has been extensively studied for multi-robot deployment. It usually takes the initial robot positions as *seeds* to the tessellation problem and then partitions the area. The logic is simple: create a frontier halfway between each robot and then stop those lines when they cross another frontier or the region's borders. We integrated in Buzz the *sweeping line algorithm*, also known as Fortune's algorithm, one of the most efficient ways to extract cell lines from a set of seeds (Fortune, 1987).

**Figure 11:**
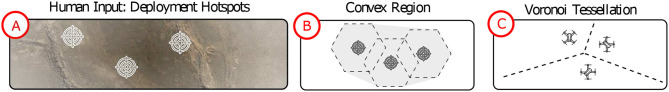
Semi-autonomous fleet deployment algorithm: **(A)** Operator hotspot inputs, **(B)** generated convex region from the hotspots list, and **(C)** the tessellation and gradient descend to uniformly cover the region.

We then cut the open cells with a user-defined polygonal boundary, shown in Figure 11: a convex region is generated from the operator hotspot inputted. At this point, each robot has knowledge of its cell's limits. For a uniform distribution of the robots in the area, we use a simple gradient descent toward the centroid of each cell, similar to the work of Cort et al. (2004). Each robot recomputes the tessellation following updates on the relative position of its neighbors; an approach that is robust to both packet loss (shown in the next subsection) and environmental dynamics. If a robot is not in the target region to be explored, a random goal inside the region is generated. Meanwhile the other members of the swarm will cover the whole region without it (larger cells). This algorithm leverages the neighbor struct (see section 2.1.1) to compute the number of seed and their relative location. It also shares the user hotspots using VS to ensure each robot has it last updated value (see section 2.1.1).

The Buzz script is available online^10^ and was extensively tested in simulations (section 3.2.2) and in the field (section 3.2.3).

#### 3.2.2. Simulations

**Figure 12:**
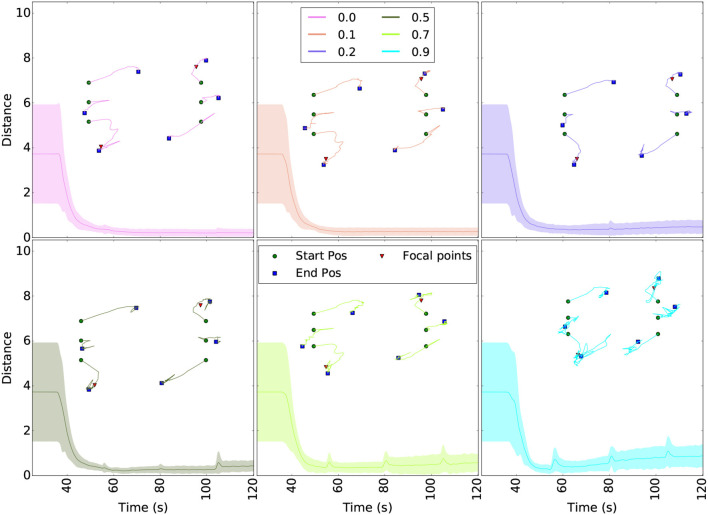
Robustness to packet drop in communication for the coverage algorithm. The curves show the distance to the ideal Voronoi cells centroids (taken as the one without packet lost) and robot trajectories for different packet drop rates. The starting positions (green dots), final formation (blue squares) and hotspots (red triangles) are plotted above the curves. Each scenario was ran 5 times and the colored area around the curves show the variability of the results. Even with 90% packet drop rate, the swarm converge to the right cell centroids.

Similar to section 3.1.2 for the first ROSBuzz algorithm demonstrated, we ran simulations to assess the robustness and scalability of the coverage algorithm. Figure 12 shows the convergence of the area coverage algorithm through a set of simulations with increasing packet drop rates.

For each of the six simulated configurations, the robots were initially distributed over two lines as illustrated with the red dots of Figure 12. The curves of Figure 12 plot the difference (euclidean distance) over time between the each robot position and their ideal Voronoi cell centroid. The ideal case is obtained from the first scenario, without any packet lost. The trajectories taken by the robots to reach their final tessellated positions are shown above the curves. The hotspots (red triangles in Figure 12) are the same for all runs and were selected in order to ensure that all six robots were already in the region to cover before launch (no random goals generated). The flat line starting each curve represents the delay before the take off command is been sent and the hotspots shared over the whole fleet. These plots demonstrate that the uniform coverage algorithm converge to the same final formation even with 90% packet drop rate. Moreover, small packet drop rates generate periodic oscillations since neighboring robots (seeds) disappear and reappear. Same reasoning apply to large drop rate: major and lasting changes in the neighbors list directly influenced the stability, but nevertheless do not prevent the fleet to converge.

**Figure 13:**
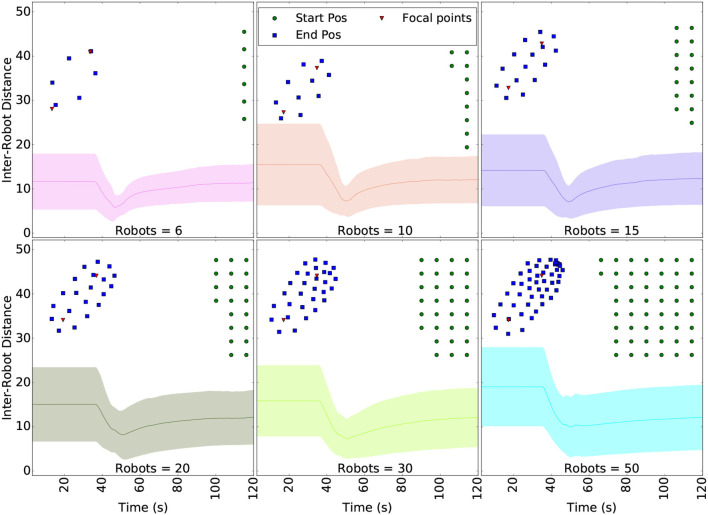
Distance between the robots while navigating to the Voronoi centroids, as the number of robots in the swarm increases.

To study the scalability properties of the coverage algorithm, we conducted another set of experiments with up to 50 robots, no packet lost, and a similar configuration as the previous simulations. Figure 13 shows the average of all pairwise distances between robots of the swarm. After the robots agreed on the hotspot values, they need to navigate toward the region to cover. This motion regroup the robots and cause a steep drop in the inter-robot distance. Upon arrival in the region, they each compute a tessellation to get their own cell centroid and navigate toward it. Close to their cell's centroid, the tessellation refined itself over time, slowly spreading the fleet over the area. The inter-robot distance slowly increases until it reaches a steady state. In the end, these results show that the software infrastructure and the algorithmic computation scales well. A simulation run with 30 robots is shown in the Supplementary Video.

#### 3.2.3. Field Deployment

The guarantee of robustness provided by the simulations above, allowed us to deploy the experiment in the field safely. The flights were conducted in an outdoor field with additional safety measures: a geofence and a velocity limit. The geofence helped to prevent the UAVs from flying too far as a result of the autonomous cells computation from arbitrary user inputs. The velocity limit make it easier for the user to understand and expect the UAVs motion.

**Figure 14:**
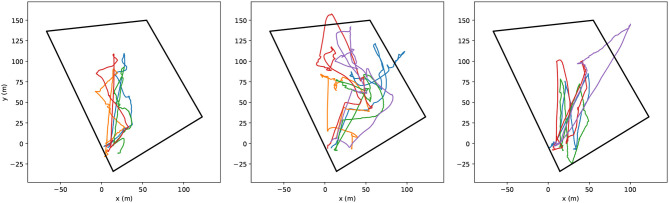
Trajectories of five UAVs in semi-autonomous exploration task following the hotspots inputted by each operator. The black polygon represents the geofence.

Three operators controlled a fleet of two M100 and three Spiris for 15 min aiming at the exploration the area comprised in the geofence limits. The resulting trajectories are shown in Figure 14. Wind burst pushed some robots out of the geofence (black polygon) on occasion. The plots tend to illustrate that the operators were using hotspots more as attracting locations for the fleet, i.e., not waiting for it to reach a stable uniformly deployed formation. Nevertheless, comments gathered from the participants indicate that they felt in control and enjoyed the experience. Where the first demonstration used pre-loaded task graphs, this one added a potential liability from its online computation of targets relying on the user inputs. The experiments still demonstrated the safe and robust software architecture, from design to simulation and field deployment. A short excerpt from the operators control is shown in the Supplementary Video.

## 4. Conclusions

This paper describes the software infrastructure ROSBuzz for the deployment of coordination behaviors on multi-robot systems. ROSBuzz integrates both the swarm-oriented programming language Buzz and the ROS ecosystem. It grants the developer of decentralized behaviors with useful swarm programming primitives and a set of essential tools for robust deployment. We described the implementation of a swarm-level barrier mechanism and an OTA update mechanism. To demonstrate the software usage, workflow and performance, we discussed the implementation of progressive task allocation strategy and a semi-autonomous exploration algorithm. Simulations for both scripts show to be robust to large packet drop rate and to scale well. To test the concept and the whole platform-agnostic infrastructure, experiments with heterogeneous teams were conducted in the field. The missions succeeded in each scenario. The task assignment experiments specifically addressed the communication performance. It was shown that throughout the whole mission, robots used less than half the available bandwidth for inter-robot communication. The semi-autonomous exploration integrated an external variable: dynamic inputs from an operator. The participants enjoyed the experiment and the fleet show robust behavior.

Based on our experience and the results of our field experiments, we are providing ROSBuzz to the robotics community: it is available^11^ online, just as the scripts described in this paper. While this paper shown robust performance from algorithm design to the field, ROSBuzz is still in early stage of development. Among the future works, the implementation of new external code (hook) must be simplified and the limitation to run with global positioning system (GPS or indoor motion capture) is currently being addressed. More laboratories have started using Buzz in their set of software tools and we hope to see the community growing. As more research will be conducted with this infrastructure, Buzz and its ROS implementation will be enhanced to further support swarm robotics field research.

## Data Availability Statement

The datasets generated for this study including the scripts used for parsing the datasets are available Online (St-Onge et al., 2020)

## Ethics Statement

The studies involving human participants were reviewed and approved by Ethical committee of Polytechnique Montreal. The patients/participants provided their written informed consent to participate in this study.

## Author Contributions

DS-O, VV, IŠ, and GB designed the architecture of the software. DS-O, VV, and IŠ implemented the software and wrote the sections of the manuscript. DS-O adapted both algorithms for the experiments and managed the experiments. VV, IŠ, and GB helped conduct the experiments and generated the plots for the manuscript. All authors contributed to manuscript revision, read and approved the submitted version.

## Conflict of Interest

The authors declare that the research was conducted in the absence of any commercial or financial relationships that could be construed as a potential conflict of interest.

## Footnotes

^1^https://github.com/MISTLab/BittyBuzz

^2^http://wiki.ros.org/rviz

^3^http://wiki.ros.org/rqt

^4^http://wiki.ros.org/plotjuggler

^5^http://the.swarming.buzz/ICRA2017/cheat-sheet/

^6^https://the.swarming.buzz/wiki/doku.php?id=buzz_examples

^7^http://www.ros.org/

^8^http://ardupilot.org/rover/

^9^https://github.com/MISTLab/ROSBuzz/blob/master/buzz_scripts/include/taskallocate/graphformGPS.bzz

^10^https://github.com/MISTLab/ROSBuzz/blob/master/buzz_scripts/include/act/states.bzz#L344

^11^https://github.com/MISTLab/ROSBuzz/
